# New Drugs, Appliances, and Things Medical

**Published:** 1892-10-15

**Authors:** 


### NEW DRUGS, APPLIANCES, AND THINGS MEDICAL.

[All preparations, appliances, novelties etc., of which a notice is desired, should be sent for the Editor, to care of The Manager, 140, Strand, London, W.C.

### UNSWEETENED CONDENSED MILK.

We have received a sample of condensed milk from the First Swiss Alpine Milk Exporting Company. This production has a distinct advantage in being free from added sugar, the excess of which debars many persons from using the usual form of condensed milk. The brand in question will keep fully three times as long as fresh milk, when the tin is opened, and as small quantities can be had this is quite sufficient for most purposes. It is not as thick as most descriptions to condensed milk, and is therefore more quickly prepared for use. Its absolute purity will recommend it to many.

### "FURFELT" CHEST PROTECTORS.

The most excellent chest protector we have yet met with is that produced by Messrs. Solport, 66, Shaftesbury Street The "Furfelt" is a combination of the purest and softes undyed lamb's wool mixed with the fur of the grey coney. The protector is so soft and silky as to be unobjectionable to the most irritable skin, and it is light and neatly fashioned. The back piece is as large as the front in the double ones, a great advantage to the delicate who are as frequently as susceptible of cold in the back as in the chest. The protectors are remarkably reasonable in price.

### THIOCAMPH DISINFECTANT.

This is a novel, powerful, and valuable disinfecting compound, which owes its invention to Dr. Reynold, the professor of chemistry in the University of Dublin. It has been known as a chemical fact, for some time, that when sulphur dioxide gas is brought into contact with camphor, a combination of the two substances ensues and a liquid is formed. From this fluid, with the addition of other volatile disinfectants, Thiocamph is made. The fluid is remarkable for holding in solution enormous volumes of sulphurous acid. Now sulphurous acid requires a pressure of at least two atmospheres to condense it from the gaseous to the liquid state. Thiocamph presents for use a fluid which nearly resembles liquid sulphurous acid in its antiseptic properties, but which is stable under ordinary atmospheric conditions. If the fluid be spread out in a thin layer, say on an old tea tray, it gives off fairly rapidly a large quantity of $SO^2$ gas. Sulphurous acid is in a moist condition a most powerful germicid e and antiseptic. Hence the value of this fluid, which has in addition the valuable and similar properties of camphor. Another consideration is this, that the gas is yielded without burning anything, and so does away with the risk of fire. After the evaporation of the fluid a crystalline mass results. If this is mixed with water a useful purifying fluid for washing floors, walls, &c., is made. We note that Thiocamph is the antiseptic fluid which is used for sanitary purposes in the Houses of Parliament. A fragrant solution in sprinkling bottles for use in the sick room is also supplied by the manufacturers—Messrs. T. Tyrer and Co., Stirling Chemical Works, Abbey-lane, Stratford, E.

